# Unusual presentation of hepatocellular carcinoma invading the anterior abdominal wall muscles

**DOI:** 10.11604/pamj.2014.17.169.3794

**Published:** 2014-03-06

**Authors:** Nayef AlShabyli

**Affiliations:** 1 Department of Radiology, Prince Sultan Military Medical City, Riyadh, Saudi Arabia

**Keywords:** Hepatocellular carcinoma, abdominal wall, metastases

## Abstract

Hepatocellular carcinoma (HCC) is a common cancer all over the world. It demonstrates a tendency for vascular invasion, producing extensive intrahepatic metastases and portal vein or inferior vena cava extension. Tumor spread of abdominal diseases via hepatic ligaments has also been reported. The author reports a rare case of HCC invading the anterior abdominal wall muscles and protruding into the subcutaneous fat.

## Introduction

Hepatocellular carcinoma (HCC) is the most common primary tumor of the liver and the third leading cause of cancer related death [1, 2]. Cirrhosis is the leading cause for development of HCC and the most common causes for cirrhosis include alcohol abuse and chronic infection by hepatitis B and C viruses. It is an aggressive tumor known for its propensity to directly invade the portal and hepatic veins, but lymphatic and distant metastases are not rare, especially in tumors greater than 5 cm [3]. The prognosis of patients with extra hepatic metastases is generally very poor. Natsuizaka et al. [4] reported a 1-year survival rate of 24,9% and median survival period of 7 months in patients with extra hepatic HCC. The most common site for metastasis includes lungs and lymph nodes followed by bones. Direct invasion of the portal and hepatic veins was reported in 29,65% and 12,54% of cases, respectively, in three large autopsy series [3, 5, 6]. Metastasis to the anterior abdominal wall has been rarely reported.

## Patient and observation

32 year old gentleman was known to have Hepatitis B virus presented to our institution complaining of right upper quadrant pain, distension, jaundice and vomiting. Laboratory findings shows mildly elevated ALT/AST and highly elevated alpha fetoprotein levels reaching 1518 ng/ml.

**Figure 1:**
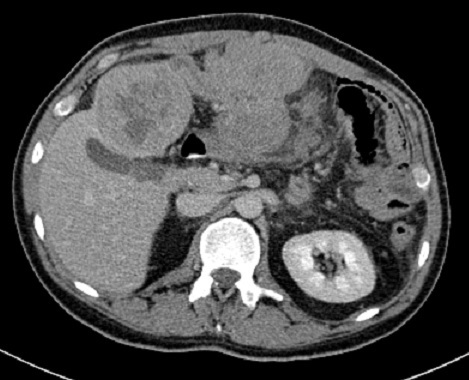
Large mass with central necrosis seen in the left hepatic mass with linear extension invading the greater momentum

**Figure 2:**
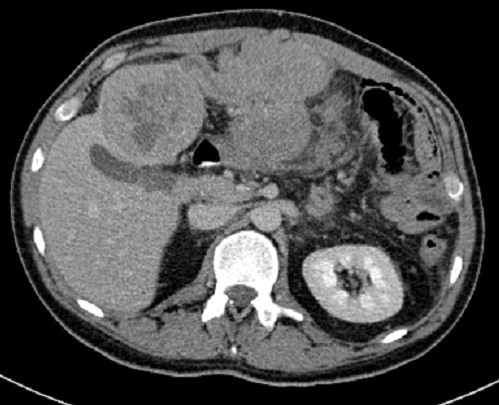
The peritoneal component of the mass is shown with loss of the fat plane between the transverse colon and the mass indicating invasion

**Figure 3:**
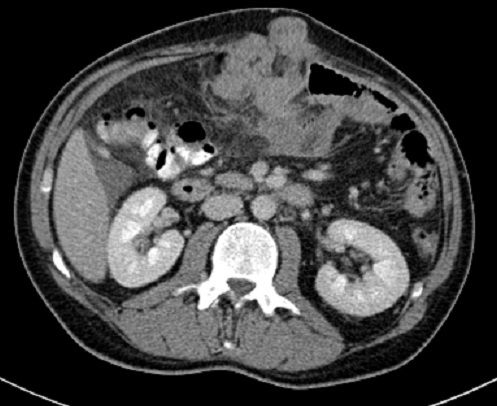
Axial view of the peritoneal component of the mass is invading the anterior abdominal wall muscles and protruding to the subcutaneous fat through linea alba

**Figure 4:**
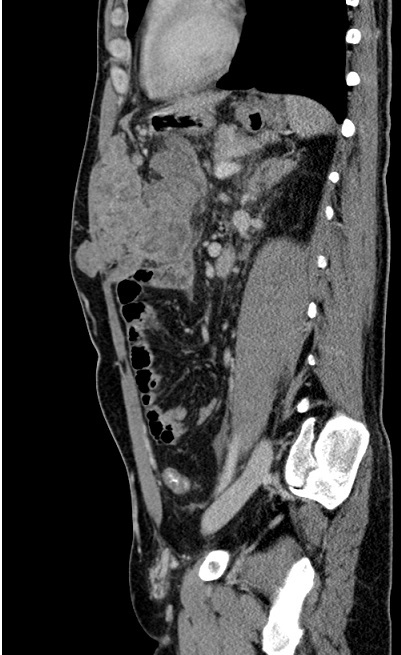
Sagittal view of the peritoneal component of the mass is invading the anterior abdominal wall muscles and protruding to the subcutaneous fat through linea alba

CT scan was performed and showed a large mass in the left hepatic lobe with area of central necrosis demonstrating peripheral isodensity to the liver and invading the greater momentum (Figure 1) with multiple conglomerate peritoneal lesions showing areas of central necrosis and indenting the transverse colon with loss of fat plane indicating invasion (Figure 2). The peritoneal component of the large left hepatic mass is invading the anterior abdominal wall and extending to the subcutaneous fat through linea alba 8 cm above the umbilicus (Figure 3, Figure 4). No other areas of metastasis is seen in the chest, abdomen and pelvis

Ultrasound guided biopsy of the liver lesion was performed and the patient developed sudden drop of hemoglobin following the procedure. CT scan performed and showed interval development of intralesional hemorrhage. After discussing the plan of palliative management with the patient, he refused to start chemotherapy and decide to be discharged against medical advise.

## Discussion

HCC is responsible for about 1 million deaths annually worldwide. The difficulty in treatment of this cancer and the reason for high mortality is the association of this cancer with cirrhosis which limits both the treatment options and increases morbidity of any modality of treatment. HCC is usually asymptomatic at early stages and has a great propensity for intravascular invasion even when the tumor is small; hence HCC is usually at an advanced stage when discovered. The most common sites of metastases of HCC include lung, peritoneum and lymph nodes. In a study by Katyal et al., tabulation of all extra hepatic metastatic sites showed the most common sites to be the lung in 81 (55%) patients, abdominal lymph nodes in 60 (41%) patients, and bone in 41 (28%) patients [7]. Rare sites of metastasis include bone and skeletal muscle. A case of HCC with adrenal gland metastasis was reported by kitagawa Y [8]. Hofmann et al. reported metastasis to the anterolateral right chest wall from HCC [9]. Coban et al. reported metastasis to the anterior chest wall in form of a left axillary mass [10]. In the study by Horita et al., bony metastasis to the sternum from HCC was demonstrated [11]. This is one of the rare instances of bony metastatic HCC. Munk et al. demonstrated metastasis in HCC to sacrum and gluteus muscles while Matsunaga et al. found extrahepatic metastasis to the distal phalanx of a finger [12] Darzi et al demonstrated abdominal wall metastasis following HCC after laparoscopy [13]. Alpha fetoprotein (AFP) usually correlates with tumor size and doubling time of AFP correlates with the tumor doubling time. Therefore AFP has a prognostic value.

## Conclusion

HCC is a highly malignant tumor that can invade and metastasize to almost any organ in the body. Therefore unusual areas of metastases should not disclose the radiologist from calling it as metastasis. Although tissue biopsy is needed in unusual cases.

## References

[1] Jemal A, Ward E, Hao Y (2005). Trends in the leading causes of death in the United States. 1970-2002. JAMA.

[2] Bosch FX, Ribes J, Díaz M (2004). Primary liver cancer: worldwide incidence and trends. Gastroenterology..

[3] Yuki K, Hirohashi S, Sakamoto M (1990). Growth and spread of hepatocellular carcinoma. Cancer..

[4] Natsuizaka M, Omura T, Akaike T (2005). Clinical features of hepatocellular carcinoma with extrahepatic metastases. J Gastroenterol Hepatol.

[5] Kaczynski J, Hansson G, Wallerstedt S (1995). Metastases in cases with hepatocellular carcinoma in relation to clinicopathologic features of the tumour: an autopsy study from a low endemic area. Acta Oncol.

[6] Nakashima T, Okuda K, Kojiro M (1983). Pathology of hepatocellular carcinoma in Japan. Cancer.

[7] Katyal S, Oliver JH, Peterson MS, Ferris JV, Carr BS, Baron RL (2000). Extrahepatic metastases of hepatocellular carcinoma. Radiology..

[8] Kitagawa Y, Tajika T, Kameoka N, Kanda Y, Watanabe T, Miura A, Teramoto T, Masai O, Onuma T (1996). Adrenal metastasis from hepatocellular carcinoma. Hepatogastroenterology..

[9] Hofmann HS, Spillner J, Hammer A, Diez C (2003). A solitary chest wall metastasis from unknown primary hepatocellular carcinoma. Eur J Gastroenterol Hepatol..

[10] Coban S, Yüksel O, Köklü S, Ceyhan K, Baykara M, Dökmeci A (2004). Atypical presentation of hepatocellular carcinoma: a mass on the left thoracic wall. BMC Cancer..

[11] Horita K, Okazaki Y, Haraguchi A, Natsuaki M, Itoh T (1996). A case of solitary sternal metastasis from unknown primary hepatocellular carcinoma. Nippon Kyobu Geka Gakkai Zassh..

[12] Otsuji M, Matsunaga S, Koga H, Kawabata N, Imakiire T, Hiwaki T, Tashiro Y, Shirahama H, Komiya S (2009). An atypical extrahepatic metastasis of the distal phalanx from hepatocellular carcinoma. Int J Clin Oncol..

[13] Nduka CC, Monson JR, Menzies-Gow N, Darzi A (1994). Abdominal wall metastases following laparoscopy. Br J Surg..

